# Paste Made from Pumpkin Seeds in the Treatment of Tænia

**Published:** 1852-11

**Authors:** 


*Paste made from Pumpkin Seeds in the Treatment of Tænia.* Translated from the French, by S. W. Butler, M. D.—We have often repeated that our indigenous materia medica is not as meagre as is made to appear, and if we employed in its study the care which we bestow upon that which commerce brings us at great expense, this truth would not need to be repeated. Here is an example. It is more than thirty years since the *Journal Universel des Sciences Médicales* described the good effects which Dr. Mongeny had obtained in cases of tænia with a paste composed of ninety grammes (about 3½ ozs.) of fresh pumpkin, and double that quantity of honey, given in three doses, an hour apart. By this means, this physician had constantly succeeded in dislodging the tænia in the course of six or seven hours, even, in many cases after all the remedies then known had failed.

This result was sufficiently important, and the innocuity of the medicine incontestable enough to have provoked an extended trial, but observations upon it have only been made by the practitioners of Bordeaux. In 1845, Dr. Brunet pointed out to his colleagues of the Medical Society, the remarkable results which he had obtained by the use of pumpkin seeds, and since that time many of his brethren have cited numerous cases of expulsion of tænia by the employment of this remedy.

At Bordeaux, the paste is prepared with forty-five grammes (about 1⅗ oz.) of the hulled seeds of the large pumpkin (*cucurbita maxima*) with the same quantity of sugar.

Here is another proof of the success of this remedy. A physician suffered almost constantly with pains in the lumbar region, and with general lassitude ; the least labor fatigued him greatly. This state had discouraged him, and made him fear an affection of the nervous system.—Having perceived that he discharged living white worms, resembling pieces of flattened dog's grass, (*chiendent aplati,*) of the length of an inch, or nearly, and although he distinguished in them a perforation, and not the slightest trace of articulation, he thought himself affected with tænia. In accordance with the advice of M. Saramea, he took, at eleven o'clock at night, thirty grammes (about an ounce) of pumpkin seeds, mixed with one-third the quantity of sugar. The next morning—twelve hours after—having taken a simple injection, this physician passed seven metres (a little more than seven yards) of tape-worm. We cite this case simply to set forth the efficacy of the means employed ; and we must here mention that, in the discussion brought forth by this fact, Dr.

Brunet said he had employed this remedy twenty-five or thirty times with success since his first communication to the Society. M. Saramea has also succeeded in a great number of cases, but he has been obliged sometimes to employ the remedy a second or even a third time. This physician cites two cases in which the root of the pomegranate, and even kousso, had failed. It is not necessary to believe, however, [in regard to the remedy in question,] that no instance of failure may occur. M. Brunet and M. Costes have pointed some out, and they have asked, with just reason, if these various results may not be consequent upon the species of tænia treated. This remedy has seemed sufficiently efficacious to warrant the Society in entertaining the proposition of one of its members, to address a note to the National Academy of Medicine, with the request to order its insertion in its proceedings, and ultimately in the *Codex.*

This communication will, we doubt not, be received favorably by the Academy, and we would hold it up as an example worthy of imitation—as occasion offers—by other societies!—*N. J. Med. Reporter.*

